# Anatomical and Physiological Differences between the White and Negro Races

**Published:** 1876-11

**Authors:** W. J. Burt


### AMERICAN JOURNAL
OF
### DENTAL SCIENCE.

Vol. X. THIRD SERIES—NOVEMBER, 1876. No. 7.

### ARTICLE I.

### *Anatomical and Physiological Differences Between the White and Negro Races.*

BY W. J. BURT, M. D.

Read before the Texas State Medical Association.

At the session of this Association, held in Austin, Texas, April, 1875, I was appointed chairman of a committee to report on the above subject.

Ethnology is divided into two principal departments—the *scientific* and *historic*. Under the former is comprised everything connected with the natural history of man and the fundamental laws of living organisms; under the latter, every fact in civil history, which has any bearing upon the question of races, their moral or physical peculiarities, or interblending of the primary divisions of the human family.

The origin of the varieties of the human races is not connected by any natural or logical necessity with their original unity. The fact of their original unity may be infallibly certain, while the time and circumstances are enveloped in mystery; for no fact in the natural world is more inexplicable than the diversity of form and color in the human race. This report, however, only contemplates the characteristic differences existing between the negro and white races.

The question at once arises, have the negro and Caucasian descended from the same parents? I answer, that history has failed to prove to the contrary, but demonstrates clearly that man is everywhere the one identical species. Profane and sacred history trace up all the nations of the earth, like streams to a common fountain and place—that fountain in the family of Noah. I am aware a few "so called scientists" have spent much time in trying to refute this statement, and have developed much ingenuity and sophistry in their efforts. But when this question is brought down to its last analysis, the origin of the negro is from one of four sources: 1st. He is a *new creation* since the flood. 2nd. He has been *changed by climate and circumstances.* 3rd. He is *developed from lower organisms;* or 4th, he is the result of God's *direct interposition.* We believe he is not a new creation; for neither history nor tradition give any account of any creation since the flood; God had finished his work sixteen centuries before it. Has climate produced this change? Carefully peruse Egyptian, Chaldean, Assyrian and Grecian histories, and search remotest antiquity with her hieroglyphic symbols; and climate, with all her intrinsic forces, has never produced this fundamental change. Run back history for one, two, yes, for three thousand years, and there is not an authenticated case.

It is but reasonable to suppose, if climate had anything to do in changing the caucasian into a negro in remote antiquity, it would continue to make them, out of such abundant material and under such favorable influences. But history and observation fail to inform us of such changes now; climate and the sun's rays may have produced a diversity of color, but it is not possible to attribute the thick lips, projecting *os calcis* and conformation of the cranium and pelvis to these influences.

The recondite causes of race, culture, moral influence and climate, all combined, have utterly failed to develop the negro and give him that philosophic disquistion and seductive eloquence, which place the white man above all created intelligence. A little common sense, oozed out through the pores of history, puts a positive denial to the statement, that climate, with all its influences, has or can so change the anatomy and physical qualities of man.

Has the negro been *developed from lower organisms?* Messrs. Tyndall, Huxley & Co., by their development theory, say that he has been evolved and developed by some intrinsic or extrusive force to his present existence, from generic elements. But these gentlemen have failed to prove by any facts that species are convertible; and that nature has powers sufficiently strong to change the generic elements and forces of the ova of one order of animals into another. They have not been able to develop a monkey, much less a man, from this prolific protoplasm.

Then, if the negro is not a new creation since the flood, and has not been developed by climate, or evolved from protoplasm, it follows that there is but one solution to the question : that this change in color and physical conformation was *caused by God's direct interposition* in the family of Noah or Ham. It is no more unreasonable to believe that God interposed, contrary to natural laws, to produce this change, just after the flood, to carry out his purpose in giving a name and a people adapted to all portions of the earth, than that he caused a flood to cover the loftiest mountains fifteen cubits high, which destroyed all living beings, except those in the Ark. This was God's direct interposition, contrary to the laws of nature. It is clearly shown by mathematical computation that, if all the waters on the earth and in it, and if all the vapour that surrounds it was condensed, the whole combined would not be enough water to produce such a flood. Yet the statement of Mose is accepted as true, and no man dares question it.

Whilst animals are of distinct species, in the different zoological provinces to which they belong, man, notwithstanding the diversity of races, constitutes but one species over the whole earth. In this respect, man appears an exception, as a being in this creation, of which he is, at the same time, the object and the end.

It is, therefore, evident that the white and negro belong to the same species, but different races, with marked peculiarities. Furthermore, these distinctions have existed through the centuries since the flood, and what the negro was in his essential attributes, in the plains of Shinar, or in the valley of the Nile, he is, in rare perfection, to-day; and stands before us, in vivid form and unique figure, a faithful representative of his race. What, then, are these differences in the two races, as they now exist? For there is a just and geometrical arrangement, and proportion of the several parts of the body of the two races, to its several destinations, offices and functions.

The physical outlines of the negro differ from the white, in his wide, flat nose, flattened temples, protruding under jaw, wedge-shaped pelvis, flat, spongy feet, protuding *os calcis*, small Gastrocnemii, convex Tibia, longer arms, as compared with the legs, woolly hair, narrow waist, widened chest, pouting lips and black skin.

Dr. Samuel P. Logan, in his osteological admeasurements of the bones of the upper and lower extremities of the two races, gives an anatomical difference which cannot possibly be influenced by external agencies. The relative length of the bones of the extremities and the relation between their proximal and distal segments are characteristic. For example, the Humerus is, relatively to the Radius, nearly one inch longer, and the Femur is, relatively to the Tibia, nearly 1½ inches longer, in the white man than in the negro; or, to express it differently, in the white man, the Humerus is nearly four inches longer than the Radius, while in the negro it is less than three inches, and the Femur nearly four inches longer than the Tibia in the white, and is only about 2½ inches longer in the negro. Another fact, developed by these measurements, is that in the white race the Fibula is longer than the Tibia, while in the negro it is shorter.

These instructive admeasurements give us this scientific fact: that the length of Tibia, compared to the length of the Femur and Tibia combined, averages about 43 per cent. in the white, and 46 per cent. in the negro; and that the length of the Radius, compared to the length of the Humerus and Radius combined is about 40 per cent. in the white, and 43 per cent in the black. So that, with two of the long bones of either extremity, we can pretty clearly designate the race to which they belong. This greater length of the bones of the distal segment is a condition approximative to that found in animals, next below man in the zoological scale. Dr. A. McDowell, during the recent war, made frequent *post mortem* examinations on the bodies of white and colored troops. He tells us that, while the chest measurements were full (up to the army requirements) he found the lungs always *lighter* and *smaller* than in the same sized chest of the white. But the most marked difference existed between the size of the liver and spleen: the liver was comparatively larger, while the spleen was much smaller, in the negro. May we not, on this fact, base the reason why the negro is less susceptible to malarious disease?

But the chief and most important anatomical distinction exists in the cranium and brain. The Caucasian skull is of an oval or elliptical shape, with no excessive prominences, and of a symmetrical form; while the negro's skull is of an oblong, compressed form, flattened laterally, the temporal muscles rising high on the parietal bones; the cranial bones harder, thicker and stronger. The prognathous skull is remarkable for the large development of the parts connected with the organs of smell.

The nasal aperture is broad allowing a larger surface for the distribution of the olfactory nerves; hence their sense of smell is more acute. Dr. S. G. Morton, has examined critically over six hundred crania of the two races, and has probably the largest collection in the world. He speaks therefore, authoritatively. He says: "The condyloid processes of the occipital bone, in the negro, are generally divided by a transverse groove into two distinct articular plates, and gives this as an evidence of their inferiority, and, by analogy, nearer relation to the lower animals' Another interesting fact is the position of the foramen magnum. In the negro, it is further back in the occipital bone, and with a less projection of the bone."

Dr. Morton took great pains to ascertain the absolute and relative capacity of the crania of the two races and of the higher order of animals. He places the average capacity of the cranium of the white man at 92 cubic inches; the average capacity of the negro at 83 cubic inches; the higher order of orang-outang and chimpanzee at from 16 to 24 cubic inches.

This difference in the cranial capacity of the two races amounts to 9 cubic inches, while the difference between the negro and highest order of animals is the astonishing amount of 59 cubic inches; which fact should forever put at rest the question of the near relationship of the negro and orang-outang.

If a line be drawn from the articulating surfaces of the occipital condyles, along the floor of the nostrils, and be intersected by another line touching the most prominent parts of the forehead and superior maxillary bone, this intersected angle is called the facial angle. This facial angle is said to be the typical expression or exponent of the relative strength of the physical and mental factors.

Taking this as true, we learn that the facial angle in the Caucasian is about eight degrees larger than in the negro, and as a consequent fact, the brain of the negro is about one-eighth less in size, weight and superficial feet. From these data we draw three important deductions:

1st. That the size and weight of the brain will be found to increase with the angle of the face to the axis of the body.

2nd. The facial angle increases in proportion to the expansion of the cranium, with a diminution of the facial bones.

3rd. The mental manifestation and power have a direct relation to the angle above indicated.

Without noticing other anatomical peculiarities, we close this part of our subject by stating that the great fault of human philosophy is its *haste* to unravel and explain, without waiting for the plodding searches of science; so that the immense extent of our ignorance, compared with that of our knowledge, has been only the more profoundly forced upon us, as discovery has advanced. The negro has little elasticity of temperament, he lacks nervous endurance and moral courage, and it is believed that he is more profoundly impressed by shock than any race on the earth; consequently he holds up badly under all surgical operations, and gives a fearful mortality to such diseases as require nervous endurance and fortitude. I will name but a few. The statistics of disease and deaths of the white and colored troops, during the recent war, complied by Drs. Otis and Woodward, show 205 deaths per 1,000 of colored troops from disease; the death rate for the same time, of the white troops, was 55 per 1,000. In typhoid fever, 1 out of every 2 8-10- died of the white, and 1 out of every 1 8-10 of the colored. In consumption, 1 of every 2½ cases died among the whites, and 12 of 13 died among the colored; and in pneumonia, 1 out of 5 among the whites, and 1 in 3 of the colored. This want of vital power and nervous endurance, I presume, results mainly from two causes, viz: The less lung capacity and smaller sized brain of the negro. The post-mortems of Dr. McDowell prove the first, and Dr. Morton demonstrates the latter; and although the liver is larger in the negro, and assists the lungs to decarbonize the blood, yet it is negative in its results, and powerless to give vital force and resistance. As a consequence of this lowered vital force and want of nervous energy, in practice, the negro cannot stand depletion, and must have stimulating and supporting treatment from the onset of disease.

The mortality from consumption in our Southern States among the negroes is extremely large. The fact that they live in a southern and temperate climate, and their habits of industry give them abundant air and exercise, should give a smaller per cent. of cases and a less mortality, if they were not more susceptible, and if they had the same vital resistance. The nature, constitution and peculiarities of the negro adapt him to the duties and work of a tropical climate, and unfit him for the cold bleak winters of the North. He, bareheaded and naked, will sleep comfortably as on a bed of roses, under a tropical sun that would kill a white man. In cold countries the negro trembles and withers in the blast, while the white man rejoices in the tempest and snow; but, in tropical regions, the former stretches forth his limbs, his eyes sparkle, and his whole person becomes alive with activity and force, while the latter files to the shade and faints from the heat. This nature also enables the negro to better resist the diseases and injurious influences endemic to hot climates; while the white man hardly dares remain, over night, on the marshes and rice plantations along our southern coast, the negro lives, works and sleeps with impunity.

The negro is peculiarly exempt from yellow and malarial fevers; his constitution is but little susceptible to the poisons that generate them. In a practice of twelve years, in a locality where malarial diseases prevailed, and where two thirds of the laboring class were negroes, and where I witnesssed twenty cases of hemorrhagic malarial fever, and knew of at least as many others, no case occurred in the negro, and he was, in a measure, exempt from the milder forms of malarious disease.

I close this report, already too long, with the statement that I have only mentioned some of the prominent differences between the Caucasian and Negro races.

